# Quantification of *Plasmodiophora brassicae* Using a DNA-Based Soil Test Facilitates Sustainable Oilseed Rape Production

**DOI:** 10.3390/plants5020021

**Published:** 2016-04-22

**Authors:** Ann-Charlotte Wallenhammar, Albin Gunnarson, Fredrik Hansson, Anders Jonsson

**Affiliations:** 1 Rural Economy and Agricultural Society, HS Konsult AB, P.O. Box 271, SE-701 45 Örebro, Sweden; 2 Precision Agriculture and Pedometrics, Department of Soil and Environment, Swedish University of Agricultural Sciences, P.O. Box 234, SE-523 23 Skara, Sweden; Anders.Jonsson@jti.se; 3 Swedish Seed and Oilseed Growers, P.O. Box 53, SE-230 53 Alnarp, Sweden; Albin@svenskraps.se; 4 Rural Economy and Agricultural Society, Borgeby Slottsväg 11, SE-237 91 Bjärred, Sweden; Fredrik.Hansson@hushallningssallskapet.se; 5 Swedish Institute of Agricultural and Environmental Engineering, Green Tech Park, P.O. Box 63, SE-532 21 Skara, Sweden

**Keywords:** *Plasmodiophora brassicae*, qPCR assay, predictive soil tests

## Abstract

Outbreaks of clubroot disease caused by the soil-borne obligate parasite *Plasmodiophora brassicae* are common in oilseed rape (OSR) in Sweden. A DNA-based soil testing service that identifies fields where *P. brassicae* poses a significant risk of clubroot infection is now commercially available. It was applied here in field surveys to monitor the prevalence of *P. brassicae* DNA in field soils intended for winter OSR production and winter OSR field experiments. In 2013 in Scania, prior to planting, *P. brassicae* DNA was detected in 60% of 45 fields on 10 of 18 farms. In 2014, *P. brassicae* DNA was detected in 44% of 59 fields in 14 of 36 farms, in the main winter OSR producing region in southern Sweden. *P. brassicae* was present indicative of a risk for >10% yield loss with susceptible cultivars (>1300 DNA copies g soil^−1^) in 47% and 44% of fields in 2013 and 2014 respectively. Furthermore, *P. brassicae* DNA was indicative of sites at risk of complete crop failure if susceptible cultivars were grown (>50 000 copies g^−1^ soil) in 14% and 8% of fields in 2013 and 2014, respectively. A survey of all fields at Lanna research station in western Sweden showed that *P. brassicae* was spread throughout the farm, as only three of the fields (20%) showed infection levels below the detection limit for *P.brassicae* DNA, while the level was >50,000 DNA copies g^−1^ soil in 20% of the fields. Soil-borne spread is of critical importance and soil scraped off footwear showed levels of up to 682 million spores g^−1^ soil. Soil testing is an important tool for determining the presence of *P. brassicae* and providing an indication of potential yield loss, e.g., in advisory work on planning for a sustainable OSR crop rotation. This soil test is gaining acceptance as a tool that increases the likelihood of success in precision agriculture and in applied research conducted in commercial oilseed fields and at research stations. The present application highlights the importance of prevention of disease spread by cleaning of farm equipment, footwear, *etc.*

## 1. Introduction

Clubroot caused by *Plasmodiophora brassicae*, is recognized as a serious soil-borne disease in Brassica crops and is associated with appreciable yield losses [1,2]. Disease outbreaks have caused problems in winter oilseed rape (WOSR) growing regions in southern Sweden and more frequently also in fields of spring oilseed rape (SOSR) and WOSR in central Sweden in recent years. This economically important disease has also proliferated worldwide in oilseed rape (OSR) and vegetable brassicas [3]. Persistent resting spores produced in high numbers remain viable in the soil for up to 17 years [4]. Integrated management strategies including the use of fungicides and resistant cultivars have been implemented in cabbage crops [5]. Chemical treatment is not an economically viable option for commercial OSR production. However, partly resistant OSR cultivars have been available to WOSR growers for approximately 10 years [6] and the release of several new cultivars onto the Swedish market has allowed WOSR production to expand into fields where *P. brassicae* is present. Soil bioassay tests were first used for monitoring the widespread prevalence of *P. brassicae* in a region in central Sweden in the 1980s [4], and were later offered and adopted as a commercial soil advisory testing service for farmers [7]. More recently developed methods with higher throughput and accuracy enabled by molecular techniques based on polymerase chain reaction (PCR) have allowed rapid assessment of the infection potential of *P. brassicae*. A protocol using real-time PCR for direct detection and quantification of genomic DNA of *P. brassicae* from resting spores in the soil has been developed and used for naturally and artificially infested soil samples containing different concentrations of *P. brassicae* [8]. A DNA-based soil testing service that identifies fields where *P. brassicae* poses a significant risk is now also available to growers [9]. The Swedish Oilseed Growers’ Association performs about 120 field experiments annually, mainly in oilseed growers’ fields. As a consequence of the recent outbreaks of clubroot [9], several field experiments have been abandoned, and thus an analysis of experimental sites for the presence of *P. brassicae* DNA has become a necessary measure prior to establishing experiments. The objective of this paper is to report the first results of implementation of the Biological Soil Mapping (BioSoM) project, including a new service for farmers and researchers based on molecular analysis of field survey samples that quickly and reliably quantifies *P. brassicae* DNA and predicts the infection potential of clubroot in field soils intended for OSR production or OSR field experiments.

## 2. Results

### 2.1. Assessment of Farm Fields

Survey results from Scania in southern Sweden in 2013 are presented in Table 1, and show that *P. brassicae* is prevalent as DNA was detected in 60% of the 45 fields on 10 of 18 the farms sampled. On one farm, DNA was detected in 8 of 11 fields sampled.

### 2.2. Assessment of Farm Fields Intended for Field Experiment on Winter Oilseed Rape

The results from sampling 59 fields in 2014, representing 36 farms in six counties, intended for field experiments in winter OSR are presented in Table 2. 44% of the fields, located on 16 farms were contaminated and in 8% of the fields *P. brassicae* DNA was found at levels >50,000 DNA copies g^−1^ soil.

The fields infested with *P. brassicae* DNA were in the counties of Scania, Halland, Kalmar, East Gothia and West Gothia, while levels below the detection limit of *P. brassicae* DNA were found in the two fields tested on the island of Gotland. On seven farms, two to five fields had to be sampled in order to find a healthy field. On five of these farms *P. brassicae* DNA was found in 75% of the fields. No *P. brassicae* DNA was found in either of the sampled fields on two farms.

**Figure 1:**
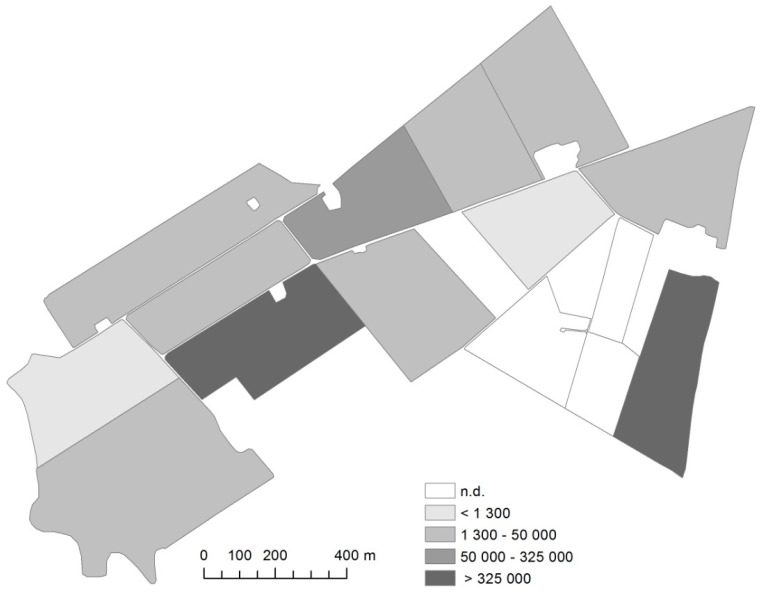
Field distribution of *Plasmodiophora brassicae* (DNA copies g^−1^ soil) at Lanna Research Station in western Sweden. n.d. represents fields with no detection.

Soil samples analyzed from 15 fields at Lanna research station (Figure 1) are illustrated by a soil map. Fields with levels below the detection level and fields with a high risk of infection and multiplication of *P. brassicae* are shown. There are only three fields (20%) below the detection level of *P. brassicae* DNA. The highest amounts of *P. brassicae* DNA, 658,750 and 823,750 DNA copies g^−1^ soil respectively, were found in fields with recent OSR field experiments.

## 3. Discussion

We here report the first results of implementation of the Biological Soil Mapping (BioSoM) project, a new service for farmers and researchers based on molecular analysis of field survey samples that quickly and reliably quantifies *P. brassicae* DNA and predicts the infection potential of clubroot in field soils.

The results from growers’ fields in the winter OSR districts of south-west Sweden show that *P. brassicae* is prevalent as DNA was detected in 60% of the 45 fields sampled. In 14% of the fields tested here (Table 1) levels >50,000 DNA copies g^−1^ soil were found, indicating a high risk of complete crop failures if a susceptible OSR cultivar is sown.

Based on previous Swedish studies [8] the following temporary guidelines have been formulated for levels of DNA copies g^−1^ soil [10]. For DNA-levels <1300 DNA copies, corresponding to approximately 3000 spores g^−1^ soil, the risk of yield loss in susceptible crops is probably less than 10% [11]. At DNA levels ranging from 1300 to 325,000 DNA copies g^−1^ soil (corresponding to 3000 and 750,000 spores g^−1^ soil, respectively) resistant cultivars are recommended. At levels >325,000 DNA copies g^−1^ soil the risk of multiplication of soil inoculum is considerable [8]. Yield loss caused by infections of *P. brassicae* depends not only on soil pre-plant DNA levels, but on other soil factors, and particularly on the time point of infection viz. on the part of growing season available after infection. Studies performed in spring OSR estimating yield loss based on soil inoculum levels assessed with bioassays showed a relationship (*R^2^* = 0.94) [1]. Recent studies on the performance of partly resistant cultivars of winter OSR confirm the variation in disease severity between years, and also point at an extremely severe yield loss of susceptible cultivars in a field experimental site with preplant inocula <10 million DNA copies g^−1^ soil [12].

The results from sampling 59 fields in 2014, representing 36 farms intended for field experiments in winter oilseed rape crop, clearly show that *P. brassicae* has also proliferated throughout the growing districts of southern and central Sweden with 44% of the fields, located on 16 farms being contaminated (Table 2). In 8% of the fields *P. brassicae* DNA was found at levels (>50,000 DNA copies g^−1^ soil) where a severe outbreak is likely to occur given optimal conditions for infection.

The presence of clubroot may cause problems at research stations where OSR field trials have been conducted over the years. The results from Lanna research station (Figure 1), clearly show an obvious spread, as there are only three fields (20%) below the detection level of *P. brassicae* DNA.

Thus, a predictive test indicating the infection potential of *P. brassicae* is essential when considering the choice of cultivar and identifying a healthy field for running field experiments. Routine testing of fields in contaminated areas is now recommended and offered by commercial laboratories [11] and the number of soil samples recently analyzed has reached a level that indicate a general adoption of this for routine clubroot detection prior to sowing. Increasing knowledge of fields where *P. brassicae* is prevalent will assist measures to prevent spread of the pathogen.

Clubroot was first discovered by assessing diseased plants in Swedish OSR crops in districts with high precipitation and a history of Brassica production in farm fields [8]. Previous findings in an extensive survey based on bioassays of 190 farm fields distributed in 18 farms where clubroot was assessed in central Sweden, showed that *P. brassicae* was present in 78% of the fields [4]. The investigations reported upon here (Table 1 and Table 2) clearly show that the pathogen has also proliferated into the main regions of winter OSR production.

A patchy distribution of *P. brassicae* has been clearly demonstrated in earlier studies [8]. Therefore, when *P. brassicae* DNA is found in a field, further information can be obtained by conducting a point sampling, e.g., collecting 10 subsamples within a radius of 3 meters at different intensities e g one sampling point per hectare, [8] to identify the variation in occurrence of *P. brassicae* DNA. At Lanna Research Station, where OSR field experiments are routinely conducted, more detailed knowledge on the spread in selected fields is necessary in order to maintain high quality in field experiments on OSR crops.

In Swedish soils with a long history of cultivation of various Brassica crops [9] the pathogen was prevalent prior to the onset of OSR production in the 1940s. Breeding for clubroot resistance in turnips and swedes started in 1929 at a branch station of the Swedish Seed Association in western Sweden, where the disease was prevalent at that time [13], and resulted in several resistant cultivars. During the late1930s experiments were carried out on infested soil at Lönnstorp experimental farm in southwest Sweden (55°66′N, 13°10′E), which is now a SLU research station. This information is of great importance in indicating that there is most likely a history of *P. brassicae* distributed throughout Sweden, and that after repeated OSR production the pathogen multiplies, as shown in the long-term field experiments in southern Sweden [14] where the OSR crop was severely diseased due to clubroot after 10–12 rotations of spring OSR grown every four years.

The important role of machinery [15] and wind [16] in the dispersal of clubroot has been demonstrated in Canadian studies identifying the importance of equipment cleaning and sanitation [15]. While inspecting a field experiment site at Bollerup in southern Sweden with very high levels of *P. brassicae* DNA (58 million DNA copies corresponding to 133 million spores g^−1^ soil), soil samples were taken from rubber boots. Samples scraped off the soles of the left and right rubber boots weighed 143.5 g and 135.5 g, respectively and contained *P. brassicae* DNA corresponding to 530 and 682 million spores g^−1^ soil, respectively. This shows that spread even by footwear might be critically significant, bearing in mind that 1000 spores g^−1^ soil is the limit of detection [9]. The potential risk of spread by footwear has to be prevented by routinely using shoe covers in experimental fields.

These results presented here suggest that *P. brassicae* is widely present in winter OSR regions and now poses a threat to OSR production in areas where clubroot was first reported only in recent years [8].

## 4. Experimental Section

### 4.1. Assessment of Farm Fields

Growers in the WOSR districts of south-west Sweden, in former Malmöhus county, were offered a soil analysis for *P. brassicae*. Analyses were performed in 2013 in samples from 45 fields on 18 farms. For some of the farms more than one field were sampled. Soil was sampled by field research staff from the Rural Economy and Agricultural Society. A soil auger with diameter 22 mm and volume 76 mL, was used to extract samples from the top 20 cm of the soil. The sampling procedure was performed according to standard procedures used by the Rural Economy and Agricultural Society in Scania, with four samples per hectare, taken within a plot of 50 × 50 m at points determined by GPS. The soil samples were sent to Eurofins Food and Agro Testing Sweden AB, Kristianstad, Sweden where qPCR analyses were performed.

### 4.2. Assessment of Farm Fields Intended for Field Experiment on Winter Oilseed Rape

WOSR trials are regularly being conducted in oilseed growers’ fields. The fields intended for WOSR production in 2014 were sampled in July prior to sowing according to the instructions for Biological Soil Mapping (BioSoM) project. They were sent to Eurofins Food and Agro Testing Sweden AB, Kristianstad, Sweden where qPCR analyses were performed.

### 4.3. Biological Soil Mapping of Lanna Research Station

At Lanna research station in western Sweden (58°38′N, 13°16′E),which is owned and run by SLU, biological soil mapping was carried out to establish a general level of infection potential for each field. The fields were sampled according to BioSoM instructions, with 40 subsamples collected with an auger as described in 4.1 along a W-shaped sampling transect covering the field. The samples were handled and analyzed according to Eurofins protocol described in [8] at the laboratory of the Department of Soil and Environment, SLU, Skara.

## 5. Conclusions

The study demonstrates that the commercially available qPCR assay offers growers and researchers a fast and reliable predictive test for determining *P. brassicae* DNA levels in individual fields and can be used as a tool in integrated OSR production and in agricultural field research. The results clearly show that *P. brassicae* (as measured by DNA level in soil) is prevalent in the main winter OSR producing regions in Sweden. Soil analysis is therefore highly recommended by the Swedish Oilseed Grower’ Association and advisory services as a management tool, and is an essential measure prior to OSR and Brassica production, research and development. Introduction of new research results in advisory work is often slow and time-consuming. However, we found that the soil test is already being adopted by commercial laboratories and by farmers.

## Figures and Tables

**Table 1:** Results of *Plasmodiophora brassicae* analyses in soil samples from 45 fields in Scania, Sweden, 2013.

DNA copies g^−1^ soil	0 *	<1300 **	1300–50,000	50,000–325,000	>325,000
Number of samples	18	6	15	4	2
% of all fields	40	13	33	9	5

* *P. brassicae* DNA not detected (number of copies below the limit of detection); ** *P. brassicae* DNA detected, but at levels below the limit of quantification.

**Table 2:** Results of *Plasmodiophora brassicae* analyses in soil samples from 59 fields intended for field experiments in winter oilseed rape in southern and central Sweden, July 2014.

DNA copies g^−1^ soil	0 *	<1300 **	1300–50,000	50,000–325,000	>325,000
Number of samples	33	16	5	2	3
% of all fields	56	27	9	3	5

* *P. brassicae* DNA not detected (number of copies below the limit of detection); ** *P. brassicae* DNA detected, but at levels below the limit of quantification.
